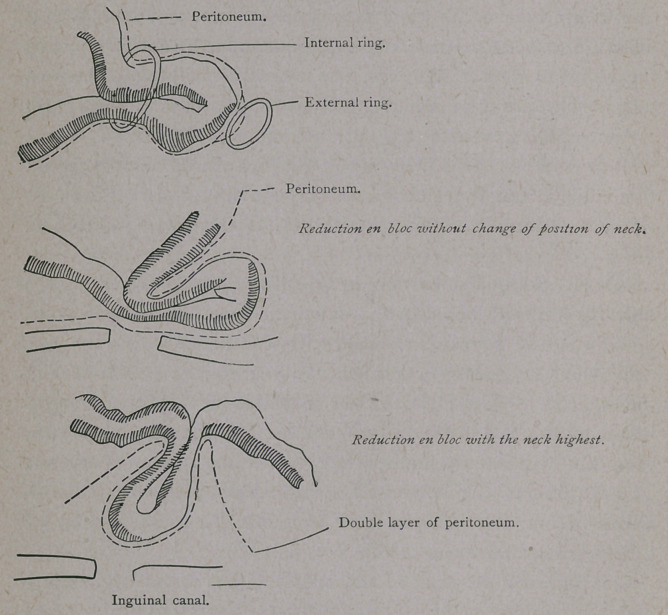# Reduction En Masse*Read before the Buffalo Medical Club.

**Published:** 1888-12

**Authors:** Herman Mynter

**Affiliations:** Professor of Surgery, Niagara University; Surgeon Sisters of Charity and Emergency Hospitals; 195 Franklin Street


### *REDUCTION EN MASSE.**

By HERMAN MYNTER, M. D.,

Professor of Surgery, Niagara University; Surgeon Sisters of Charity and Emergency Hospitals.

Among the rare accidents which may occur during taxis for a strangulated hernia, the false reduction (reposition en bloc, en masse) is about the rarest. I remember during my student days to have seen such a case, and three weeks ago I met one here in Buffalo, and operated it successfully. The patient, a German grocer, fifty-four years of age, had for the last twenty years suffered from left inguinal hernia, which was retained by a truss.

* Read before the Buffalo Medical Club.

It often came out, but was generally reduced with ease. On October 22d, in the evening, after lifting a heavy weight, the hernia appeared and soon became painful. During the night, vomiting and increasing pain in the belly occurred. Dr. Congdon was called and found a protrusion as large as a goose-egg in the upper part of the left scrotum, outside the inguinal canal. He tried moderate taxis, without anesthesia, but without result. Next morning I was called in consultation by Dr. Congdon. By the examination no scrotal hernia was seen. The inguinal canal was large, patulous, and free, and behind the inner opening of the inguinal canal the finger could feel a round, elastic, smooth tumor, which gave an impulse on coughing. Above Poupart's ligament a circumscribed tumor, as large as a goose-egg, was felt by deep pressure, also giving an impulse on coughing. There was clear percussion over the tumor. The patient had himself tried taxis during the night, but had not discovered that the scrotal tumor had disappeared. Vomiting had continued without yet being of fecal nature, and the pain in the belly was increasing. Pulse ninety-six. Hot fomentation was tried for a few hours, but, as the symptoms of strangulation steadily increased, herniotomy was performed at four o'clock P. M., Drs. Congdon and Eugene Smith assisting. An incision, three inches long, was made above Poupart's ligament, in the direction of the inguinal canal. The external ring was found soft, patulous, and normal. The canalis inguinalis was thereafter opened by incision of its different muscular layers, and the hernial sac found behind the inner opening of the canal, between the fascia transversa and peritoneum. The sac was caught hold of with a forceps and with the greatest ease pulled out of the wound, opened and found to contain, besides a large amount of serous exudation, a coil of intestine about five inches long, intensely congested but otherwise in fair condition. The neck of the sac was the constricting point; it was callous and tense, and did not admit the tip of a finger. It was incised with the herniotome, the bowels were reduced, and the whole sac was thereafter dissected out, ligated with catgut near the ring, and cut off.

The inguinal canal was closed with six deep, twisted, silver sutures, cut off short, and a short drain left in connection with the stump of the sac below the lowest suture. The superficial wound was closed with catgut. One mishap occurred: by removing with scissors some shreds of tissue in the wound, the vas deferens was cut over high up, the upper end disappeared, and could not be found.

The further course was favorable. Vomiting ceased immediately, the bowels moved spontaneously on the third day, and the whole wound healed by first intention without a drop of pus. No particular trouble was observed from the severing of the vas deferens except a very moderate epididymitis.

Very little is said in the larger English works on surgery on this subject, but the German works contain quite lengthy articles on reductio spuria, especially Bardeleben, and Billroth & Pitha.

Dr. R. Schmidt mentions, in Billroth & Pitha's surgery, three different forms of reductio spuria, which have one thing in common, that the hernia is reduced with the sac, or within the sac, while the strangulation continues and is produced by the neck of the sac. In the first form, the real reduction en bloc, the strangulation takes place in the neck of the sac and the sac and its contents are reduced by taxis, while the strangulation continues. The whole sac is torn loose from its connections and reduced, either behind the external ring (interstitial inguinal hernia) or totally behind the internal ring.

In that case the neck of the sac is either found highest up, with the fundus near the internal ring, (as happened in my case,) or the neck continues to be near the internal ring, while the fundus may deviate towards the navel, pelvis, or the iliac fossa. The parietal layer of the peritoneum must follow the neck of the sac so that it covers the whole sac like a funnel, when the neck is found torn loose and reduced high up. There is then a double cover of peritoneum. Reduction en bloc is found both in small and recent, and in old and large ruptures. Both inguinal and femoral hernias may be reduced en bloc.

The connective tissue around the sac seems to be stretched more than torn, as suggilations are not generally found. On the other hand, the incision does not fall in the scrotum, but over the inguinal canal, which accounts for the author's failure to mention whether, as a general rule, the sac is torn loose and, therefore, a large bleeding cavity found in the scrotum or not.

*INTERSTITIAL HERNIA.**

*Reduction en bloc without change of position of neck.*

*Reduction en bloc with the neck highest.*

In the second form, found only in inguinal hernia, the contents of the hernia are by taxis reduced into a cavity formed by dilatation of the inguinal canal. The sac itself is not dislodged, and the result is, therefore, not a reduction en bloc, but simply a reductio spuria.

In the third form, which is only found in femoral hernia, the protrusion is dislodged laterally, but not reduced into the abdominal cavity. On account of the shortness of the femoral canal

* From Billroth & Pitha.

an interstitial reduction cannot occur, and the hernia is found between the adductor longus and pectineus muscles. It is only possible in very fat patients. The fascia covering these muscles is ruptured by the taxis.

One other form, mentioned by different authors, deserves notice. By very violent taxis the sac may rupture, and the coil may be reduced through the tear (and become strangulated by it) between the abdominal muscles, or between them and the fascia transversa, or between the fascia and the peritoneum. The tear is found near the neck, oftener on the posterior side. The whole sac has been found torn off and the coil reduced, though still strangulated by the neck. Bardeleben mentions still a number of subdivisions of reductio spuria, but they are all, it seems to me, included in the above divisions.

The cause of reduction en bloc and reductio spuria is a faulty taxis, either too violent or in a faulty direction. A violent taxis has been used, particularly in those cases in which a cavity has been formed between the different integuments on the femur, or in cases of rupture of the sac. A faulty taxis is used when the whole protrusion is pressed up in the direction of the abdominal cavity before trying to diminish its size by compression, kneading and reduction of some of its contents, or by not keeping the direction of the canal in view during taxis. That is the reason why patients often manage to produce this reduction themselves. The diagnosis is not difficult, particularly if seen early, when the tumor can be felt distinctly above Poupart's ligament. If the examination be delayed, the increasing meteorismus may quickly disguise the tumor. The fundus of the sac may often be felt distinctly behind the internal ring, giving an impulse on coughing. If the mishap has occurred for the surgeon, he will have discovered that the tumor possibly disappeared and recurred several times during taxis, that no gurgling was felt, and no relief produced.

The symptoms continue in spite of the seemingly successful reduction and a careful examination will reveal what has happened. What is to be done? It is recommended to let the patient try by walking, straining, and coughing, to reproduce the hernia again, and if he succeeds careful taxis must be done, or herniotomy performed. If the patient fails in this, prompt action is necessary. The herniotomy, which then is nearer a laparatomy, is done along the inguinal canal. If the fundus of the sac is met, as in my case, it is pulled out and then opened in the usual way. If the body or the neck is met, the sac, which then often is double, is opened in situ. In either case the neck may have to be incised before the bowels can be returned. In all cases the sac ought to be extirpated near the neck, and the inguinal canal closed with twisted silver sutures. I have done this in a number of herniotomies and in cases of radical cure for hernia, and never yet have I had reason to regret it. Around the silver sutures a plastic infiltration takes place, so that some weeks after the operation a hard plate-like formation is felt around and beneath the scar, surrounding and enveloping the silver sutures, and although it, in course of time, disappears more or less, we may continue to feel increased hardness and string-formed horizontal adhesions where the wires are buried. I consider this form of radical cure for hernia the most successful and, under proper antiseptic precautions, the most innocent and surest method.

195 Franklin Street.

## Figures and Tables

**Figure f1:**